# Coarse-graining as a downward causation mechanism

**DOI:** 10.1098/rsta.2016.0338

**Published:** 2017-11-13

**Authors:** Jessica C. Flack

**Affiliations:** Santa Fe Institute, Santa Fe, NM 87501, USA

**Keywords:** collective computation, endogenous coarse-graining, regularity estimation, organizational levels, biological effective theories

## Abstract

Downward causation is the controversial idea that ‘higher’ levels of organization can causally influence behaviour at ‘lower’ levels of organization. Here I propose that we can gain traction on downward causation by being operational and examining how adaptive systems identify regularities in evolutionary or learning time and use these regularities to guide behaviour. I suggest that in many adaptive systems components collectively compute their macroscopic worlds through coarse-graining. I further suggest we move from simple feedback to downward causation when components tune behaviour in response to estimates of collectively computed macroscopic properties. I introduce a weak and strong notion of downward causation and discuss the role the strong form plays in the origins of new organizational levels. I illustrate these points with examples from the study of biological and social systems and deep neural networks.

This article is part of the themed issue ‘Reconceptualizing the origins of life’.

## 1. Introduction

Downward causation is among the most controversial and obscure concepts in evolutionary biology. Simply stated it is the idea that higher-level features can be a cause of behaviour by lower-level components [1,2] (see also [3–7]). On the one hand, this seems self-evident. Governments make laws and laws constrain individual behaviour, more or less forcing individuals to act in certain ways—stopping at a stop sign, for example. There are many similar colloquial examples to be found in the biological and social sciences. However, as soon as one spends a little time considering how this causality works trouble arises.

To summarize a long and convoluted debate (a bibliography: https://philpapers.org/browse/downward-causation), downward causation suffers from the criticism that it is not materialist. As the argument goes, higher levels of organization are ‘just’ temporal and spatial patterns that are the outcome of dynamics at a lower level. As patterns, they have neither material instantiation nor agency and hence cannot be causes. Of course this criticism suffers itself from a criticism: the ‘turtles all the way down’ problem.

Here I suggest we can reconcile this conflict by being operational and examining how adaptive systems identify regularities in evolutionary or learning time and use these perceived regularities to guide behaviour. I propose we move from simple feedback to downward causation when components tune behaviour in response to estimates of coarse-grained, aggregate properties. Elsewhere I have suggested that for tuning to be possible the coarse-grained variables need to have a slow time scale compared with (i) the microscopic behaviour producing them and (ii) the computation used to estimate them [8–10]. I will make these ideas concrete with examples from the biological and artificial intelligence literatures, but before I do, there are some additional issues worth emphasizing.

Adaptive systems generally are collective—meaning that there are multiple, semi-independent components making decisions and contributing to system dynamics [11]. It is not necessarily (it is perhaps rarely) the case that all components perceive similarly the aggregate properties describing the system or tune to them in the same way. This heterogeneity in tuning to aggregate properties is particularly likely in adaptive systems in which learning plays a big role. Hence a useful distinction, which I will come to in a moment, is between what we might call weak and strong forms of downward causation. Recognizing that adaptive systems tend to be intrinsically heterogenous, noisy, and collective brings into focus the reason why this tricky concept is worth arguing about—in its strong form, as I will illustrate through an example, it locally reduces entropy and creates order, which facilitates extraction of energy to do work.

I use the term *apparent downward causation* when tuning is partial and imprecise. In this weak or minimal form, only a few components need to be tuning their behaviour to estimates of aggregate properties for there to be some downward causation in the system. Apparent downward causation does not demand that estimates of aggregate properties be correct or even good predictors of the system’s future state or successful strategies (although that would be useful). Furthermore, components do not need to agree in their estimates of the variables. To reiterate, what matters is just that some components are tuning. This allows exploration of how the strength of the downward causation changes as a function of system dynamics and contributes to system performance.

Apparent downward causation becomes *effective downward causation*—the strong form—when [8–10]:

— aggregate properties are predictive of the future state of the system (slow variables);
— aggregate properties are robust to small perturbations;
— estimates of these variables are used nearly universally by all components to tune decision-making;
— components largely agree in their estimates of these variables; and
— as estimates converge there should be an increase in mutual information between the microscopic behaviour and the macroscopic properties.

As with apparent downward causation, under effective downward causation, components need not be in agreement about how to best to tune to these variables. The degree of agreement will depend on whether decision-making—in learning or evolutionary time—is influenced by other types of heterogeneity in the system (for example, resource heterogeneity), which by influencing cost may place strong constraints on component decision-making, and also on robustness-adaptability tradeoffs. High agreement—high homogeneity—in strategy would likely make the system sensitive to perturbations and move it towards a critical point, which can have costly robustness implications [12].

Notice that by operationalizing apparent and effective downward causation as described above, we avoid violating the materialist requirement because the components are tuning to patterns they perceive and hence are ‘doing the work’. I will not discuss in this paper when the operational concept of downward causation I am proposing conforms to strict definitions of causality (e.g. [13,14]). However, I will discuss (§6 how the causal contributions of the environment and collective behaviour to the aggregate properties can be roughly quantified, and I will propose that from an operational perspective it makes sense to start talking of a new level of organization when the criteria for effective downward causation are met. I explore this further in §7. Because the conceptual framework I am developing here rests in part on the concept of coarse-graining, in §2, I give a brief review of coarse-graining in physics and introduce the concept of endogenous coarse-graining–coarse-graining performed by the system (as opposed to a scientist) as an inference mechanism [8–10]. In §3, I show through an example how coarse-graining can produce effective downward causation. In §6 I discuss challenges, focusing in particular on how the collective and noisy character of adaptive systems impact coarse-graining and downward causation.

## 2. Coarse-graining: general properties

The second law of thermodynamics states that the entropy of the Universe is increasing [15]. In apparent contrast to the second law, matter and radiation appear have started out in thermodynamic equilibrium—a high entropy state [16,17]. In addition, the gravitational field at the birth of the Universe was highly structured (low entropy yet also smooth and featureless) but is becoming less ordered [18]. These two facts coupled to confusing use of the terms, ‘entropy’, ‘structure’, ‘order’, ‘uniformity’, etc. have led to a variety of at odds or unclear positions on what it means to say that entropy is increasing. As I am by no means an expert on this topic, I, with gratitude to Sean Carroll, refer the reader to Wallace [17], who nicely summarizes the conventional thinking and space of positions. He offers a restatement in which he emphasizes that the early Universe was not at global thermal equilibrium, only local thermal equilibrium, and gives the reasons why.

For our purposes here, the important point is that entropy is increasing and, as it does, at least some of the detailed information we initially had about the system is lost, making prediction about the behaviour of the system difficult. My interest in this is twofold. First, a point which i will get to in a moment, is how we can improve our capacity to make predictions under this condition. Second, a point which I get to in §3 is how we can reduce entropy locally to increase capacity for prediction locally.

Additionally, classical fluctuations at the microscopic scale—small, fast-timescale (non-directional) changes in component behaviour—can make reliance on microscopic behaviour for macroscopic predictions difficult. And, relying on the microscopic behaviour for prediction can require specifying many details. Ideally, we would like our prediction to be based on a compact description of system behaviour.

For these reasons, to make good predictions we can move to what is called a coarse-grained description in which some of the microscopic detail has been smoothed out. To make this more concrete, think about temperature [19].

Temperature is the average speed of particles in a system. Temperature is a coarse-grained representation of all of the particles’ behaviour—the particles in aggregate. When you know the temperature you can use that to predict the system’s future state better than you could if you actually measured the speed of an individual particle. Temperature can predict the future state of the system (assuming it is at equilibrium) more computationally efficiently because it requires fewer degrees of freedom.

This is why coarse-graining is so important. It gives us what is called in physics and biology the basis for an effective theory. An effective theory allows us to model the behaviour of a system without specifying all of the underlying causes that lead to system state changes. Effective theories by definition are agnostic to system mechanics. Some effective theories are even at odds with the mechanics—for example, the Bardeen–Cooper–Schrieffer (BCS) theory of high temperature superconductivity (discussed in [20]). The effective theories of interest here are those that have a basis in mechanism but nonetheless ignore many of the details.

Having a basis in mechanism is a critical property of a coarse-grained description—it is ‘true’ to the system. It is a simplification of the microscopic details. In principle, a coarse-grained description does not introduce any outside information to the subset of microscopic interactions over which it is performed (but see for examples of technical exceptions [21,22]). This ‘lossy but true’ property is one factor that distinguishes coarse-graining from other types of abstraction.

A second property of coarse-graining is that it is an integration over component behaviour. An average is a simple example but more complicated computations are also possible, as discussed in §3. I emphasize this property of integration to distinguish coarse-graining from other forms of regularity extraction like such as loss-less compression in which noise and redundancies are removed from the dataset but where the compressed output is not itself the result of an integration.

Normally when we talk of coarse-graining, we mean coarse-grainings that we as scientists impose on the system to find compact descriptions of system behaviour sufficient for good prediction. However, we can also ask how adaptive systems identify regularities and build effective theories to guide adaptive decision-making and behaviour [8–10,23]. To distinguish coarse-graining in Nature from coarse-graining by scientists, we call coarse-graining in Nature *endogenous coarse-graining* [8] (see also [24,25]). I now go through an example of endogenous coarse graining in an animal social system. To give a sense of the range of applicability of this concept I also very briefly discuss related concepts in molecular biology and neural networks.

## 3. Endogenous coarse-graining and downward causation: an example

Our coarse-graining example comes from the study of the dynamics producing power structure in a well-study macaque society model system. The review below is brief, serving to illustrate a few critical points. For further important detail, I refer the reader to [9,10].

In this system power is operationalized as the degree of consensus among group members that an individual can win fights (see [26] and references therein). The degree to which the group perceives an individual capable of winning fights is encoded in a network of subordination signals. An individual sends a subordination signal during peaceful interactions to a receiver it perceives, through a history of fighting, as likely to win fights—fighting is necessary because temporally stable factors such as experience and size of the alliance network, not just body size, influence fight outcomes. Subordination signals formalize agreement to the subordinate role in a dominance relationship, which is a kind of proto-contract. The signal is unidirectional (nearly always emitted by the same individual until the underlying asymmetry grows small or is reversed) and emitted in peaceful settings rather than during conflicts so it is less likely to be perceived by the receiver as meaning only submission in the current conflict.

Subordination signals are only emitted when the sender perceives a large asymmetry in fighting ability in its opponent’s favour. In other words—when it perceives that the cost of continued aggression is greater than the cost of subordination (in this case, signalling is the optimal strategy [27]). The decision to send the signal is the output of an individual level computation. The computation involves integrating over a history of fight outcomes in order to compute an estimate of the magnitude of asymmetry. The subordination signal serves as a coarse-grained representation of the history of fight outcomes over some period.

Fighting continues after the subordination contract has been formalized through signal exchange but at a reduced rate. This provides a mechanism by which the relationship might reverse in the future and is roughly analogous to a ‘background process’ running on your computer.

Individuals appear to roughly track the number of signals they have received from particular individuals, as well as the identity of signal senders. An individual computes its power—the degree to which it is collectively perceived as capable of using force successfully in fights—by coarse-graining the network of status signalling interactions (algorithms described in [26]) to compute the collective perception. Hence the aggregate variable individuals are estimating here is the *degree of consensus* in the group that they can win fights. The power distribution (DSP) results from the collective computation by all individuals of their power scores.

Whereas the dominance relationship formalized by subordination signal exchange is a course-grained representation of fight outcome history, a power score is a course-grained representation of the signalling network, which encodes an individual’s fighting ability as collectively perceived by the group. The direction of signal exchange and the power score both constitute for each individual effective theories that respectively predict, in the case of the dominance relationship and the power score, social tolerance from the signal receiver and the average cost of social interaction. Finally, in case it is not obvious it is worth noting that the collective computation in this example has two phases—an *information accumulation* phase in which the individuals, like sensors, gather information semi-independently about who is capable of winning fights, and a consensus or *aggregation phase*, in which that information is shared in a signalling network that encodes the power distribution [26,27] (also applies in neural systems [28]).

Coarse-graining, which happens both during the information accumulation phase and also during information aggregation phase of the collective computation, is advantageous for at least two reasons. One reason is that coarse-graining allows individuals to make predictions without requiring they indefinitely store all of the details of their interactions. Once they have signalled, the time series of fights leading up to the signal can (in principle, we do not yet know exactly how this works) be deleted from memory. Going forward individuals only need retain the signal, which summarizes the history of fights leading up to signal exchange, and the outcome of any new fights. Hence coarse-graining may allow *memory minimization*.

A second reason coarse-graining is advantageous is it allows individuals to identify slowly changing regularities that may not be evident in the fine-grained social interactions if these fluctuate for transient and/or contextual reasons, as fights do. Coarse-graining by smoothing out noise improves predictions. Hence coarse-graining results in *uncertainty reduction through slow variable construction*. An important subtlety is that even though the coarse-graining is lossy, from a functional perspective information is *gained* because the signal to noise ratio is higher and the uncertainty about what constitutes a ‘good’ strategy decreases. There is not yet a good language for discussing this transition. This point may be similar to the idea that in the normal derivation of an arrow of time, coarse-graining over microstates loses information, but information is gained about the arrow of time (anonymous reviewer 2017, personal communication).

Both the accuracy with which the DSP reflects true fighting abilities and the skewness of the DSP are functionally important. A DSP will be a reliable predictor of interaction cost if it has mutual information with the underlying (and not directly observable) distribution of fighting abilities and changes slowly [27]. The right skewness of the DSP influences conflict management. For example, heavy-tailed distributions make otherwise costly conflict-management strategies, such as policing, accessible to individuals who occupy the tail of the distribution of power. The reason for this is that individuals in the tail are perceived to be disproportionately powerful and are unchallenged when they intervene.

The system exhibits apparent downward causation when the DSP has consolidated enough so that it changes more slowly than the microscopic fight behaviour producing it and at least one individual uses estimates of its power scores or other properties of the DSP, like skewness, to make predictions about interaction cost and tune behaviour. The system begins to show effective downward causation when/if (i) the collectively estimated DSP is robust to small perturbations in signalling patterns and has a slower time scale than the fight outcome network (many fights per hour with a per individual time scale on the order of hours [29]) underlying it and the signalling network used to compute it, (ii) the DSP predicts the cost of social interaction, (iii) individuals’ estimates of properties of the DSP begin to converge—in other words over time individuals come agree through learning and increasing sample size who is powerful and who is weak and roughly by how much and (iv) the mutual information between DSP and the underlying distribution of fighting abilities is high—meaning that DSP is capturing regularities in the system.

With respect to (iii), the probability of convergence on similar estimates increases as the fight outcome network becomes fully connected—meaning all individuals have fought each other, as individuals come to have similarly sized datasets on fight outcomes, when there is social learning (correlated learning), when there is disparity in fighting ability—in other words, when regularities can be easily learned and are costly *not* to learn—in other words, *when individuals have an incentive to accurately estimate regularities* [27], when the computational capacity of components is similar, and as a result of positive feedback induced by the downward causation itself.

There is also simple effective downward causation at the pairwise level as a result of the subordination signal exchange. In this case, signal exchange (i) strongly predicts for signal sender tolerance by the receiver around resources and so reduces uncertainty (increases confidence in the prediction of cost) about the cost of social interaction with the signal receiver and (ii) because the signal is unidirectional and emitted by the individual perceiving itself to be weaker, it strongly reduces uncertainty about the state of the dominance relationship for *both* individuals in the pair. The implications of these two properties are that sender and receiver reference the signal exchange for decision-making and tune their behaviour as they deem appropriate given their respective roles in the relationship. In practice, this means that signal senders spend greater time in proximity to signal receivers than in the absence of signal exchange. Signal exchange improves the quality of the social relationship.

## 4. Coarse-graining in other adaptive systems

Endogenous coarse-graining has also been described in molecular systems, where Fontana and co-workers have referred to it as internal coarse-graining [24,25]. The motivation for this work is as follows. In molecular biology there is a huge space of possible protein–protein interactions, particularly in the case of cellular signalling. One approach for dealing with this problem of combinatorial complexity is the brute-force approach: explicitly enumerate every possible combination of molecular species. This is not only computationally intractable but produces a space of protein–protein interactions that is much larger than the actual space we observe in Nature and leaves us with an unsatisfying and incomprehensible description that needs to be simplified through tedious, and often non-biologically principled, dimension reduction.

A second approach has been to identify rules that take into account the contexts in which a particular protein–protein interaction is actually observed and then use these rules in simulation to study how different cellular phenotypes are produced. This also has disadvantages, reviewed in [24], which include that the higher-level protein–protein ‘units’ identified by this approach may again be units that the system does not recognize or cannot use. Fontana and co-workers amend this approach by adding the requirement that the only allowed molecular patterns are those that are at the lowest resolution the system can handle. Fontana and colleagues call these units ‘fragments’. Fragments are molecular interaction patterns that are independent or non-overlapping and, critically, are patterns that the system recognizes and uses. They are sufficient higher-level descriptions of system dynamics.

This work provides a second example of how thinking about coarse-graining from the point of view of the system itself can help us understand the key causal features generating its macroscopic behaviour. It is not yet clear however whether this work has anything to say about downward causation. And an important difference between the social system work described in §3 and here is that the aggregate properties in the primate case result from *collective coarse-graining by individuals within the system*. The work on internal coarse-graining in molecular systems so far has not addressed how molecular fragments are produced. Related ideas have been explored in [30–33].

## 5. Coarse-graining and compression in deep neural networks

Finally, in machine learning and neural network studies, coarse-graining and compression by neural nets have been proposed as mechanisms by which neural nets can learn high-level representations of data. Although the emphasis in this paper is on endogenous coarse-graining—coarse-graining performed by the system rather than imposed by scientists—I include discussion of these deep learning results because increasingly the distinction between a neural network as a method for doing prediction versus a neural network as an artificial intelligence that does its own prediction, is eroding.

Although neural networks are built by humans they are typically built through a kind of trial and error process with little principled understanding (so far) of how they work. Only recently has a concerted movement developed to figure out whether, when viewed through the correct lens, the design solutions different groups are converging on reflect more general information processing and collective principles based in information theory, Bayesian inference and physics (e.g. [34]).

The papers I briefly discuss here suggest the answer is yes and interestingly (to me at least!) identify principles similar to those discussed earlier in this paper: (i) multilayer systems with each layer consolidated through coarse-graining and effective downward causation, such that the coarse-grained output one layer up, influences the microscopic behaviour one (and in some cases two layers) down through downward causation, thereby reducing uncertainty and (ii) in which good coarse-grained representations of the microscopic behaviour are collectively computed by components over two phases—an information accumulation phase and an aggregation phase.

The first paper was written by Mehta & Schwab [35]. Mehta and Schwab begin by pointing out that deep learning includes a large number of neural net architectures, some of which use many layers of artificial neurons to learn important descriptive features from training data. These deep neural networks, or DNN’s, out perform shallower networks with as many parameters. Why this is the case remains an open question. Mehta and Schwab showed that the DNN architecture is essentially an iterative coarse-graining procedure. Each layer takes as input the coarse-representations of the data computed by the layer below and performs its own coarse-graining on that input. This successive feature extraction procedure enables the DNN to learn the relevant, high level features in the data and, consequently, to generalize to out of sample data. Mehta and Schwab showed that there is an exact mapping between variational renormalization group in physics and the DNN architecture via restricted Boltzmann machines. The show in their numerical simulations that the DNN discovers iterative coarse-graining through self-organization. One complication is that renormalization works best on data with many symmetries and increasingly machine learning methods are being applied to unstructured or weakly structured data. Two open questions are whether deep learning can offer insights into how coarse-grain on data that does not have a fractal structure (a machine learning/physics question) and whether effective downward causation in adaptive systems can increase symmetries at the microscopic level making the system as it consolidates increasingly amendable to coarse-graining (my question).

The second paper was written by Schwartz-Ziv & Tishby [36], building on the work of Tishby & Zaslavsky [37] and the work of Tishby *et al.* [38] in 2000, and it is on what is called in information theory the ‘information bottleneck’. I mention this paper second because Tishby made the connection between the information bottleneck and deep learning in part by reading the paper of Mehta & Schwab [39]. The information bottleneck is a learning optimization procedure that compresses the input data with the goal of retaining only the most general features useful for prediction and getting rid of the rest—the ‘noise’. In a serious of experiments, Schwartz-Ziv and Tishby showed that the neural networks they tested converged on the information bottleneck, meaning the DNN’s extracted all relevant information and suggesting that the DNN’s may be using this kind of optimization process. Schwartz-Ziv and Tishby’s experiments also suggested that the optimization performed by the DNN’s in their experiments was characterized by two phases—a fast *fitting phase*, during which DNN learns to label or classify its data, and a slow generalization or *compression phase*, during which it learns to apply the labels correctly to new data. This is similar in principle to the finding (described in §3) for the monkey case study and for neural decision-making [28] that collective computation is characterized by two phases—an information accumulation phase—essentially fitting—and an aggregation or consensus phase—essentially generalization. One important difference is that in our work the fitting phase is slow and the generalization phase is relatively fast.

It seems a promising idea that coarse-graining and compression via either the information bottleneck or some form of variational renormalization, more generally, may underlie the capacity of DNN’s to generalize. It is extremely interesting that very similar, and possibly the same processes, may account for the origins and consolidation of multi-scale temporal and spatial structure in adaptive systems, as illustrated by the example in §3 and treated at greater length in a paper on life’s information hierarchy [10]. There are many exciting, open questions including whether how broadly the principles of fitting and compression, or information accumulation and aggregation, apply. Next I enumerate some challenges, focusing on properties of adaptive systems that make coarse-graining and effective downward causation non-trivial. I leave it to others to determine if these challenges also apply to neural networks.

## 6. Challenges and predictions

**Figure 1.**
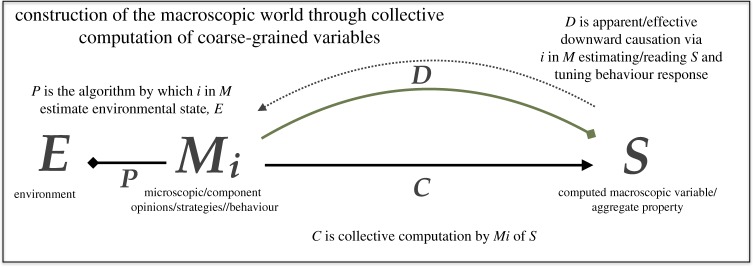
Schematic illustrating collective computation of macroscopic properties through coarse-graining and the mechanics of effective downward causation. (Online version in colour.)

Because adaptive systems are imperfect information processors coarse-graining in Nature is unlikely to be a ‘true’ simplification of the microscopic details as it is the physics sense. An open question is how good do the coarse-grainings need to be to be useful predictors—meaning, more useful than simply relying on a rich description (many parameter model) of the the microscopic behaviour or immediate state of the environment? This raises a thorny set of issues. Does it make sense to talk about a ‘correct’ coarse-graining when the components performing the computation are error prone and have finite data and computational capacity? There are many issues to unpack here. To help make the issues concrete, consider figure 1.

In figure 1, *E* is the state of the environment. Mi are the strategies/behaviour/opinions of the microscopic components about *E* (and *S*, see below), *S* is the coarse-grained, aggregate variable. *P* is the algorithm that maps the environment onto component behaviour in *M*. *C* is the collective computation that aggregates the information in *M* and outputs *S*. *D* is the algorithm that captures both how the components, *i*, in *M* estimate *S* and use the estimate to tune their behaviour.

Operationally, we can proceed by asking how well a collectively computed coarse-grained variable *S* recovers the value of *S* as computed by an observer with perfect information about the regularities in the system or environment. The idealized value of *S* is important in so far as it provides a benchmark for assessing how much information processing efficiency could be improved. Comparing the collectively computed variable to the idealized also allows us to consider whether there is indeterminacy in the system that is detectable by the observer but not the components. By calculating the conditional mutual information for these variables we can determine the contribution of the environment/some underlying constraint versus the collective computation to *S*. In such cases where the environment or an underlying constraint contributes only a small amount, we can conclude that [40]—components are effectively *constructing their macroscopic worlds* through collective behaviour and information processing ([10], see also [41] for a more fundamental version of this idea applied to physical systems). Power in the monkey case study is an example of a macroscopic variable that is loosely tied to a ground truth (underlying fighting ability, which has to be inferred) and is partly the outcome of collective dynamics. Stock price is another example—it is loosely tied to a firm’s intrinsic value and also the outcome of collective dynamics. In general, I expect that when information processing is very important, collective computation will largely be responsible for *S*. I further predict that macroscopic variables resulting from this process will scale superlinearly. By contrast, in systems with strong energetic constraints, I expect macroscopic properties will have high conditional mutual information with the environment or underlying constraints and macroscopic variables will scale sublinearly, as they do in the case of mass and metabolic rate [42].

In addition to using information theory to determine whether the aggregate variable is largely constructed or, alternatively, mirrors the exogenous environment or some underlying constraint, we can also quantify the extent to which a new organizational level is consolidating out of *S* by asking if the *I*(*S*;*M*|*E*) is increasing over time. If the *I*(*S*;*M*|*E*) is increasing over time, this suggests that the system is overcoming or at least minimizing the inherent subjectivity resulting from relying noisy components with finite computational capacity and constrained or partial information. How could this be happening?

Time scale separation is one possibility. If the time scale associated with a coarse-grained or aggregate variable is very slow compared to the microscopic behaviour producing it, it effectively becomes the ‘social’ environment (i say ‘social’ here to distinguish it from the exogenous environment, *E* we started with) and components can tune to it regardless of whether it actually does a good job of summarizing the regularities in *E* or now in *M*—think of social institutions that are highly constraining and very hard to change or the power distribution in the monkey case study. Time scale separation between *M* and *S* facilitates learning and hence coordinated and organized behaviour thereby minimizing the consequences of subjectivity due to noisy components with different windows on the world. Of course it seems likely the system will fail at some point if the aggregate properties components rely on for prediction do not capture the regularities in *M* or *E*—failure may just take longer if the time scale separation is very large. In the monkey case study, both the signalling network and power structure have a much slower time scale than the fight dynamics.

A second possible mechanism for minimizing inherent subjectivity is to make estimates of regularities in *E* and *M* a collective process. This information aggregation can be as simple as pooling many different estimates of *E* and/or *M* and taking the average, or it can involve more complicated collective computations as in the monkey power case (discussed in [26], see also [43] for importance of network structure to the computation). Either way the idea is that each component contributing to the computation of the estimate will have independent or semi-independent estimates based on having been exposed to different subsets of data or states of the environment and possibly having a different computational capacity. This is a variant of a wisdom of crowds type argument. The fact, however, that integrating over many estimates can yield a better overall estimate is a somewhat trivial insight and not, in my mind, where the interesting issues lie.

Interesting questions concern the details of the aggregation portion of the collective computation itself (see also [43]). In other words, what are properties of the collective computation allow it to produce ‘good’ estimates [11,26,27,44]? Is the computation additive or is it non-decomposable into the individual estimates? Are contributions redundant or is there heterogeneity in component contributions? How sensitive is it to perturbations, bias and gaming, and individual and group? This issue of information aggregation–how estimates (and also strategies and decision rules) combine to produce aggregate properties has been tackled a bit in the consensus formation and web search literatures (e.g. [26]), food web literature (e.g. [45]), in the social circuits literature [8,46], developmental circuits literature (e.g. [47]) and the financial markets (e.g. [48] and forecasting literatures (e.g. [49], but is largely untouched). It is, however, an important issue as collective computation is a way to overcome subjectivity in information processing yet also may, as a result of higher order interactions and synergies, produce aggregate properties that bare little relation to the environmental states or underling constraints they are supposed to summarize.

A final issue worth mentioning is that there are a large number of algorithms/procedures by which components in adaptive systems could be coarse-graining or compressing data during the information accumulation/fitting phase. These algorithms are explored in a wide range of subdisciplines within the cognitive neuroscience literature (e.g. [50–52]) but generally focus on the computations underlying decision making and the role of memory and Bayesian inference. Although the possibility that there may be generic principles of inference and collective computation is appealing, it is perhaps not that interesting if the space of algorithms for doing information accumulation and aggregation is degenerate.

## 7. Conclusion

The basic idea proposed in this paper is that as a consequence of integrating over abundant microscopic processes, coarse-grained variables provide better predictors of the local future configuration of a system than the states of the fluctuating microscopic components. In doing so, they promote accelerated rates of microscopic adaptation [8]. Slow variables facilitate adaptation in two ways: they allow components to fine-tune their behaviour and free components to search at low cost a larger space of strategies for extracting resources from the environment. This phenomenon is illustrated by work on metabolic reaction networks [53], the formation of dominance contracts [54] and the power-in-support-of-conflict-management example reviewed in §3, and by work on the role of neutral networks in RNA folding [55]. In the RNA case, many different sequences can fold into the same secondary structure. This implies that, over evolutionary time, structure changes more slowly than sequence, thereby permitting sequences to explore many configurations under normalizing selection (e.g. [55]).

As an interaction or environmental history builds up at the microscopic level, the coarse-grained representations of the microscopic behaviour consolidate, becoming for the components increasingly robust predictors of the system’s future state. We speak of a new organizational level with effective downward causation when [8,10]:

— the estimates are (functionally) good approximations of the idealized aggregate properties, summarizing regularities in the system well enough to be useful for prediction;
— the aggregate properties are sufficient to predict system dynamics at the macroscopic scale;
— the system’s components rely to a greater extent on the coarse-grained descriptions of the system’s dynamics for adaptive decision-making than on microscopic behaviour;
— the course-grained estimates made by components are largely in agreement; and
— there is mutual information between the consolidating higher level/layer and the microscopic behaviour one level down.

Notice that I am proposing that the consolidation of a new level of organization requires, in addition to downward causation (§1), that component estimates of aggregate properties, either individually or collectively, are functionally good approximations of the aggregate properties as computed by an observer with perfect information about the regularities in the system. The idea is that convergence on ‘good-enough’ estimates underlies non-spurious correlated behaviour among the components, but an open and system dependent question is what constitutes ‘good enough’. This in turn leads to an increase in local predictability and drives the construction of levels of organization—what I have referred elsewhere to as the information hierarchy [10]. To be clear the increase in correlation is due to the components increasingly perceiving the world in the same way—an effect that amplifies itself through effective downward causation. However, this does not necessarily imply the absence of heterogeneity. Heterogeneity in strategy will remain in the system as components might not be in agreement about how best to tune to the perceived aggregate properties—even when they agree in their estimates of these variables. This can depend on whether decision-making is influenced by other types of heterogeneity, such as variation in resource holding potential, and on robustness-adaptability trade-offs, as high agreement/homogeneity in strategy use would likely make the system sensitive to perturbations and move it towards a critical point [12,56,57].

The probability of estimate convergence should increase as sample size grows, if the computational capacities of the components are similar, and through an amplification process caused by the consolidation of new levels of organization as apparent downward causation becomes effective downward causation. The result is the slow variables become fundamental macroscopic properties that are a public good of sorts (for a similar concept in the study of markets, see [58,59]) in so far as they reduce uncertainty about the state of the system for all components and so allow components to more appropriately refine strategies.

Downward causation need not be a mystical concept if we think operationally about how biological systems identify regularities and use these to tune behaviour.

In my view two key questions for twenty-first century biology are the precise mechanisms by which Nature coarse-grains and how the capacity for collective coarse-graining influences the quality of the effective theories that adaptive systems build to make predictions. Answering these questions might help us gain traction on some traditionally quite slippery philosophical questions. Among these, what are the natural scales of adaptive systems and once we have a principled means for detecting these scales will we find regularities that suggest adaptive systems like physical systems can be described by laws [42,60,61]?

## Data accessibility

This article has no additional data.
